# Repetitive negative thinking in adolescence: a mixed methods study

**DOI:** 10.1186/s13034-025-01005-0

**Published:** 2025-12-10

**Authors:** Nikki N. Huang, Michelle L. Moulds, Jill M. Newby, Aliza Werner-Seidler

**Affiliations:** 1 School of Psychology, Faculty of Science, University of New South Wales, Kensington, NSW 2033 Australia; 2 Black Dog Institute, Randwick, NSW 2031 Australia

**Keywords:** Repetitive negative thinking, Rumination, Worry, Co-rumination, Adolescence, Qualitative, Mixed methods

## Abstract

**Background:**

Adolescence is a critical window for preventing common mental disorders. Repetitive negative thinking (RNT) is a transdiagnostic process contributing to depression and anxiety and a key prevention target. However, little is known about adolescent experiences of RNT, especially during early adolescence. Furthermore, co-rumination, the interpersonal analogue of RNT, remains underexamined. This study characterised diverse facets of adolescent RNT and co-rumination in a self-selected community sample with elevated depression and anxiety symptoms, incorporating adolescent and parental perspectives.

**Methods:**

A mixed‑methods online survey was completed by 72 adolescents (mean age = 14.51 years; 69% female, 10% non‑binary), including 26 younger (aged 10–14) and 46 older adolescents (aged 15–18), and 57 parents (adolescent mean age = 12.25 years; 44% female). Participants answered open- and close-ended questions regarding key aspects of adolescent RNT, and completed standardised measures of adolescent RNT, co‑rumination, depression, anxiety, wellbeing, and quality of life. Quantitative data were analysed descriptively; qualitative data were analysed using thematic coding.

**Results:**

Both younger and older adolescents reported comparable levels of elevated RNT, co-rumination, depression and anxiety, moderate wellbeing, and low-to-moderate quality of life, broadly consistent with parental reports. Qualitative responses were mostly consistent across adolescent age groups. Adolescents showed comprehensive understanding of worry but low conceptual familiarity with rumination, especially among younger adolescents. RNT was primarily past-focused, verbal, and most likely to occur at night, interfering with sleep. Older adolescents reported slightly longer and more frequent RNT episodes than younger adolescents. Across both age groups, common RNT triggers included being alone or in a quiet environment, interpersonal/social, and school-related triggers. Younger adolescents were less able to identify specific RNT triggers. Common RNT-reduction strategies included distraction and interacting with others. However, almost half of adolescents were unsure what triggered their RNT and reported not doing anything to stop it. Most adolescents and parents perceived RNT as entirely unhelpful or purposeless. RNT reduction was expected to improve emotional wellbeing and engagement in valued actions.

**Conclusion:**

RNT and co-rumination are commonly experienced in adolescence. RNT causes distress to young people often lacking in effective coping strategies and warrants further attention alongside co-rumination as transdiagnostic targets in preventive mental health programs.

**Supplementary Information:**

The online version contains supplementary material available at 10.1186/s13034-025-01005-0.

## Background

Adolescence is a period of heightened vulnerability for common mental health challenges [1, 2]. The onset of puberty, transitions between primary to secondary schooling, and shifting relationships and self-concepts are just some of the many stressors that young adolescents face [3, 4]. Against this backdrop, the frequent emergence of anxiety and depressive symptoms during early adolescence (ages 10–14; [3]) is unsurprising [2, 5, 6]. Left unaddressed, these symptoms can develop into common mental disorders by mid-to-late adolescence. Recent epidemiological studies suggest that the peak age of onset is 14.5 for social anxiety disorder and obsessive-compulsive disorder, 15.5 for generalised anxiety disorder and panic disorder, and 19.5 for depressive disorders [2]. Interventions aimed at preventing anxiety and depression should be delivered prior to these peak vulnerability periods for disorder onset.

Given the frequent co-occurrence of depression and anxiety and their numerous overlapping risk factors [7, 8], transdiagnostic prevention approaches make clinical and economic sense [9]. A promising target for transdiagnostic preventive interventions is repetitive negative thinking (RNT), the common process of getting stuck in one’s own negative thoughts without arriving at useful insights or solutions [10]. Broadly defined as a thinking style that is recurrent, negatively valenced, and difficult to control, RNT encompasses rumination (repetitive thoughts about past negative events and emotions) and worry (repetitive thoughts about potential future negative outcomes) [11]. RNT predicts not only depression and anxiety symptom severity and comorbidity, but also the development, maintenance, and relapse of depressive and anxiety disorders in both adolescents and adults [12–16].

Growing recognition of RNT as a promising target in the prevention and treatment of depression and anxiety has led to the development of interventions that specifically aim to reduce RNT. Rumination-focused cognitive behavioural therapy (RFCBT; [17]), its subsequent adaptations [18, 19], and interventions that have incorporated RFCBT principles (e.g., [20, 21]) train adolescents and adults to identify when they are engaging in RNT and to shift towards more helpful thinking processes or other emotionally-adaptive behaviours. RNT-focused interventions have demonstrated effectiveness in both preventing and reducing depression and anxiety [22–26], further supporting the choice of RNT as a transdiagnostic preventive intervention target. However, existing RNT-focused preventive interventions typically target mid-to-late adolescence (e.g., [19]: ages 15–22; [18]: ages 18–24), developmental periods that coincide with or are subsequent to peak ages of onset for depression and anxiety disorders [2]. There is an absence of RNT-focused preventive interventions for adolescents under the age of 15, the developmental period when depressive and anxiety symptoms are likely to first emerge [23].

This prevention gap warrants attention, since RNT has been repeatedly identified as contributing to depression, anxiety, and various other mental health challenges in young adolescents [27–29]. RNT may be more malleable during the heightened neural and social plasticity of early adolescence [30, 31], and developing RNT-reduction skills may not only prevent depression and anxiety, but also improve functioning and wellbeing. These views have been echoed by young people with lived experience of depression and anxiety, who emphasised the importance of early RNT-focused interventions (“the earlier the better”; [23]).

To create effective and engaging RNT-focused preventive interventions, we must first develop a comprehensive understanding of how adolescents experience RNT. A mixed methods approach, integrating both quantitative and qualitative methods, provides complementary perspectives to elucidate psychological lived experience [32–34]. Existing research typically investigates RNT using standardised self-report questionnaires developed by researchers and clinicians. Although such quantitative methods allow for efficient measurement and facilitate comparisons, they should be complemented with qualitative methods for a more nuanced and in-depth understanding of psychological phenomena [33, 34]. The small number of qualitative studies investigating individual experiences of RNT in older populations have provided valuable insights [35–38]. Specifically, participants across these studies described RNT as overwhelming and difficult to control, often triggered by negative emotions or social interactions, and frequently managed using distraction or interpersonal support, albeit with limited success. These insights have been gathered from clinical or treatment-seeking older adolescents or adults, but the phenomenological lived experience of RNT in early adolescence remains unexamined. Given the distinct context of early adolescence, including developing cognitive capacities, self-awareness, and unique sociobiological stressors [3, 4], a mixed methods study incorporating early adolescent perspectives is important to elucidate not only the prevalence and severity of RNT, but also the subjective meanings and contexts characterising RNT in this developmental period. Furthermore, the qualitative experience of RNT across adolescence has been underexplored. Capturing adolescent lived experience of RNT using both quantitative and qualitative approaches can inform the development of age-appropriate interventions, ensuring the language, examples, and strategies resonate with populations they are being delivered in, to facilitate program engagement.

In addition to RNT, the adolescent experience of co-rumination, which involves excessive discussion of personal problems, also warrants further investigation. Co-rumination typically occurs in close relationships in which individuals repeatedly rehash issues without reaching new insights or solutions, a relational pattern that can foster closeness but also increase negative social interactions and distress [39]. Co-rumination predicts increased depressive and anxiety symptoms, and more severe depressive episodes over time, including in early adolescence [40–42]. As an understudied transdiagnostic phenomenon, co-rumination is an important companion construct to RNT that merits further investigation and potential intervention in adolescence.

In light of these considerations, the current study aimed to investigate adolescent experiences of RNT and co-rumination using a combination of qualitative and quantitative methods via an online survey, with inclusion of previously underexamined early adolescent perspectives. As the first mixed methods study in this area, we decided to recruit a 10-18-year-old sample from the community, but to categorise the sample into younger (10–14-year-old) and older (15–18-year-old) adolescents. This allowed us to explore whether there are important developmental differences between how younger and older adolescents experience RNT. To incorporate valuable parental insights, parents of adolescents aged 10–18 from the community were also recruited to participate in our online survey. The survey included open-ended and multiple-choice questions regarding personal definitions, triggers, content, frequency, and duration of adolescent RNT, as well as perceived RNT purpose and coping strategies. To characterise the nature of the sample and measure co-rumination, participants also completed standardised self- or parent-report questionnaires about this construct.

## Methods

### Participants

The sample was comprised of adolescents and parents of adolescents aged 10–18. Participants were recruited via social media advertisements and the Black Dog Institute website. Adolescents had to be aged between 10 and 18 and parents had to have an adolescent aged between 10 and 18 to participate in the study. Apart from age and/or being a parent of a 10–18-year-old, no other inclusion or exclusion criteria were applied. For adolescents aged 10–14, parental consent for participation was obtained. Participants were given the option of completing surveys as a parent-adolescent dyad, whereby the parent and their adolescent child completed separate surveys, but their responses were linked. Alternatively, adolescents could opt to participate without their parents doing the same and vice versa. In return for participation, participants were given the opportunity to enter prize draws to win one of three $100 gift cards or one of three $50 gift cards.

### Procedure

All parts of the study were completed online on the Qualtrics platform. Participants read the Participant Information Sheet and Consent Form (PISCF) online before providing informed consent. For adolescents aged 10–14, parental consent for participation was obtained, and adolescents read a Participant Information Sheet explaining study participation conditions in age-appropriate language. Additionally, all adolescents were required to complete a three-item Gillick Competency Task correctly to demonstrate sufficient understanding before proceeding to the measures. Participants then completed study questionnaires (see Measures section below). Adolescents were asked to respond to questions about themselves while parents responded to questions about their child. This study was approved by The University of New South Wales Human Research Ethics Advisory Panel (Approval Number 7162).

### Measures

#### Demographic information

Participants were asked to provide basic demographic information, including age, school grade, gender, country of birth, primary home language, disability, neurodivergence, and mental health history (i.e., current/prior mental health diagnoses, pharmacological and/or psychological treatments).

#### Standardised measures

*Revised children’s anxiety and depression scale-25 (RCADS-25)* The RCADS-25 is a 25-item measure of anxiety and depression, validated for use in children in Grades 3–12 (ages approximately 8–18) with good reliability [43, 44]. Adolescent and parent participants completed the self-report and parent-report versions of the RCADS-25 respectively. Internal consistency in the current sample was α = 0.91 and 0.88 for the adolescent- and parent-report versions respectively.

*Perseverative thinking questionnaire—child version (PTQ-C)* The PTQ-C is a 15-item transdiagnostic measure of RNT. The PTQ-C has been validated for use in children aged 9–15 with excellent reliability [45]. Internal consistency in the current sample was α = 0.93.

*PTQ-C adapted parent-report version* Since validated parent-report versions of the PTQ-C have not yet been published, we created a bespoke questionnaire to assess adolescent RNT from a parental perspective. Our 6-item questionnaire was adapted from items 4, 5, 8–11 of the PTQ-C [45], and these items were chosen for adaption as they were deemed possible for parent-report (see Additional File 1). Internal consistency in the current sample was α = 0.91.

*Co-rumination questionnaire—short form (CRQ-SF)* The CRQ-SF is a 9-item measure of co-rumination between friends, validated for use in children aged 11–17 with good reliability [40]. The CRQ-SF was administered to adolescent participants only. Internal consistency in the current sample was α = 0.89.

*Co-rumination questionnaire—parent-adolescent (CRQ-PA)* The CRQ-PA is our adaptation of the 8-item Co-Rumination Questionnaire—Mother-Adolescent (CRQ-MA), which assesses co-rumination between mothers and their adolescent children [46]. The CRQ-MA has been validated for use in adolescent children and their mothers [46]. We retained the 8 items from the CRQ-MA but adapted the adolescent-report version to ask respondents about co-rumination with their mother or father (“whichever parent you usually talk to about your problems”) for a more inclusive questionnaire (See Additional File 2). The parent-report version of the CRQ-MA was administered without modification as the original wording was appropriate for use by either mothers or fathers. Internal consistency in the current sample was α = 0.92 and 0.81 for the adolescent- and parent-report versions respectively.

*Short Warwick-Edinburgh mental well-being scale (SWEMWBS)* The SWEMWBS is a 7-item measure of mental wellbeing, validated for use in children aged 10–16 with good reliability [47, 48]. The SWEMWBS was administered to adolescents only, since a parent-report version of this measure is not available. Internal consistency in the current sample was α = 0.80.

*Pediatric quality of life inventory—short form (PedsQL-SF)* The PedsQL-SF is a 15-item measure of quality of life across four subscales: physical, emotional, social, and school functioning, validated for use in children aged 5–18 with good reliability [49]. Adolescent and parent participants completed the self-report and parent-report versions of the PedsQL-SF respectively. Internal consistency in the current sample was α = 0.85 and 0.83 for the adolescent- and parent-report versions respectively.

#### Bespoke RNT questionnaires

Several open-ended and closed questions were adapted from an adult questionnaire [35] to investigate adolescent experiences of RNT from both adolescent and parent perspectives. This measure included questions asking young people to share their personal definitions of worry and rumination, how often they engage in RNT and for how long, the typical things they think about when worrying and/or ruminating, what triggers RNT, how they cope with it, as well as the perceived purpose and benefits of RNT. Adolescent- and parent-report versions of this bespoke questionnaire can be found in the Additional File 3.

### Data analysis

#### Qualitative data analysis

Descriptive statistics and frequencies were calculated to describe and characterise the sample using SPSS v30.

### Quantitative data analysis

A thematic analysis approach was taken to analyse the qualitative data [50]. One author (NH) coded responses to open-ended questions from the Bespoke RNT Questionnaires using a flexible combination of deductive- and inductive-dominant approaches [51].

The coding process began with NH reading through participant responses to each question and developing a codebook to categorise the data. This process was repeated for each open-ended question. Initial response categories were then reviewed and refined by AWS and MM. Once response categories were finalised for each question, NH coded whether a participant’s response fell into any of the response categories for the corresponding question using binary coding. Participant responses to a particular question could fit into more than one response category. For ambiguous responses, AWS and MM were consulted and the item was coded based on mutual agreement.

Reflexivity is a key element of qualitative analysis [50] and all researchers involved in the coding process critically reflected on the potential influence of their assumptions and experiences when developing response categories and coding the data. The research team comprised of three females with diverse research and clinical experience in mental health. NH is a provisional psychologist with research interests in adolescent RNT. AWS and MM are researchers and clinical psychologists with extensive experience in repetitive negative thinking and adolescent mental health.

## Results

### Sample

**Fig. 1:**
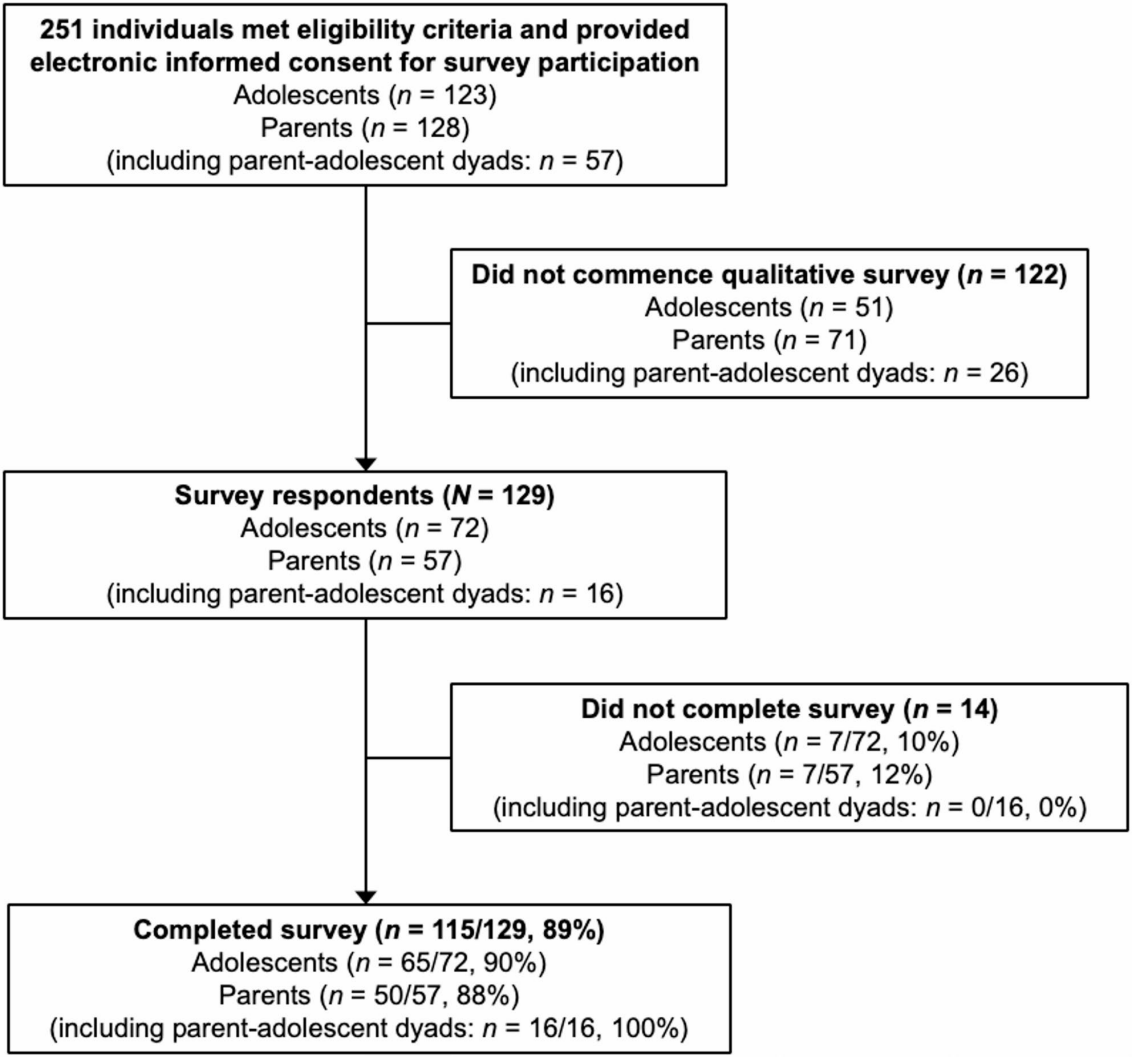
Participant flow diagram

Figure 1 summarises participant flow through the study. A total of 123 adolescents and 128 parents met eligibility criteria (including 57 parent-adolescent dyads) and provided electronic informed consent to participate. Following consent, 51 adolescents and 71 parents (including 26 parent-adolescent dyads) did not commence the qualitative RNT survey and were therefore excluded from data analysis, giving a total of 72 adolescent and 57 parent (including 16 parent-adolescent-dyad) survey respondents. Of these survey respondents, a total of 65 adolescents and 50 parents (including 16 parent-adolescent dyads) completed the entire survey. Participants who did not complete the survey did not provide reasons for non-completion. Since some survey questions were not answered by all participants, response frequencies were calculated as a proportion of the total number of participants who responded to a particular question, rather than as a proportion of the total number of survey respondents.

### Participant characteristics

#### Adolescent-report sample

As shown in Table 1, the majority of adolescent participants were female (69%) and 10% identified as non-binary, another gender, or preferred not to say. Participants had a mean age of 14.51 (SD = 2.55), with 36% aged 10–14 (hereafter: younger adolescents) and 64% aged 15–18 (hereafter: older adolescents). Most participants were born in Australia (93%).

The majority of participants reported a current or prior mental health diagnosis, with the most common diagnoses being generalised anxiety disorder (46%) and depression (40%). Almost three quarters of participants reported current or prior experience of psychotherapy (74%), and a sizeable proportion reported current or prior mental health medication use (42%). The majority of participants (69%) identified as neurodivergent, with the most common forms of neurodivergence being ADHD (31%) and autism (25%).

**Table 1:** Participant characteristics (adolescent-report sample: *N* = 72)

*Age*		*Current/prior psychological therapy*, *n (%)*	
Mean (SD)	14.51 (2.55)	Yes	53 (73.61)
Median	15	No	19 (26.39)
Range (Min – Max)	10–18	*Current/prior mental health medication*,* n (%)*	
*Gender*,* n (%)*		Yes	30 (41.67)
Female	50 (69.44)	No	42 (58.33)
Male	15 (20.83)	*Neurodivergence*,* n (%)*	
Non-binary	5 (6.94)	Attention deficit hyperactive disorder	22 (30.56)^1^
Use a different term	1 (1.39)	Autism	18 (25.00)
Prefer not to say	1 (1.39)	Specific learning disability	6 (8.33)
*Country of birth*,* n (%)*		Tourette’s or other chronic tic disorder	3 (4.17)
Australia	67 (93.06)	Intellectual disability	1 (1.39)
United Kingdom	2 (2.78)	Unsure	15 (20.83)
Fiji	1 (1.39)	None of the above	27 (37.50)
Pakistan	1 (1.39)	*Self-report measures*,* Mean (SD)*	
South Africa	1 (1.39)	RCADS-25 total^2^	34.04 (12.74)
*Current/prior mental health diagnosis*,* n (%)*		Depression subscale	14.89 (6.07)
Generalised anxiety disorder	33 (45.83)	Anxiety subscale	19.15 (7.97)
Depression	29 (40.28)	PTQ-C^2^	39.00 (10.67)
Attention deficit hyperactive disorder	18 (25.00)^1^	CRQ-SF^2^	21.06 (7.51)
Social anxiety disorder	14 (19.44)	CRQ-PA^2^	19.59 (8.10)
Eating disorder	9 (12.50)	SWEMWBS^2^	18.40 (2.97)
Obsessive compulsive disorder	9 (12.50)	PedsQL-SF^2,3^	51.09 (16.92)
Panic disorder	5 (6.94)	Physical functioning subscale^3^	64.63 (23.73)
Separation anxiety disorder	4 (5.56)	Emotional functioning subscale^3^	39.83 (20.40)
Post-traumatic stress disorder	4 (5.56)	Social functioning subscale^3^	55.85 (28.90)
Conduct disorder	1 (1.39)	School functioning subscale^3^	38.81 (27.74)
Bipolar disorder	0 (0.00)		
Schizophrenia	0 (0.00)		
Unsure	5 (6.94)		
None of the above	22 (30.56)		

^1^Discrepant percentages in self-reported ADHD in the Current/prior mental health diagnosis and Neurodivergence sections of this table may be due to differences in question wording. To assess current/prior mental health diagnosis, participants were asked: “Have you ever been diagnosed by a professional (e.g. a doctor or psychologist) with any of the following? Pick all that apply”. To assess neurodivergence, participants were asked: “Do you identify as neurodivergent? Pick all that apply.”

^2^CRQ-SF: Co-rumination Questionnaire—Short Form; CRQ-PA: Co-Rumination Questionnaire—Parent-Adolescent; PedsQL-SF: Pediatric Quality of Life Inventory—Short Form; PTQ-C: Perseverative Thinking Questionnaire—Child Version; RCADS-25: Revised Children’s Anxiety and Depression Scale-25; SWEMWBS: Short Warwick-Edinburgh Mental Well-Being Scale

^3^Reverse-scored

#### Parent-report sample

As shown in Table 2, parent-report data were collected with regard to a more even distribution of female (44%) and male (56%) adolescent children. The children of parent participants had a mean age of 12.25 (SD = 2.06), with 88% aged 10–14 and 12% aged 15–18. Most adolescents were born in Australia (96%).

The majority of the parent participants’ adolescent children were reported to have a current or prior mental health diagnosis, with the most common diagnoses being generalised anxiety disorder (39%), attention deficit hyperactivity disorder (30%), depression (14%), and social anxiety disorder (14%). Additionally, sizeable proportions of adolescents were reported to have current or prior experience of psychotherapy (68%) and/or pharmacotherapy (41%).

**Table 2:** Participant characteristics (parent-report sample: *N* = 57)^1^

*Age*		*Current/prior psychotherapy, n* (%)	
Mean (SD)	12.25 (2.06)	Yes	39 (68.42)
Median	12	No	18 (31.58)
Range (Min–Max)	10–18	*Current/prior mental health medication*,* n (%)*	
*Gender*,* n (%)*		Yes	23 (41.07)
Female	25 (43.86)	No	33 (58.93)
Male	32 (56.14)	*Parent-report measures*,* Mean (SD)*	
Non-binary	0 (0.00)	RCADS-25^2^	25.54 (10.53)
Use a different term	0 (0.00)	Depression subscale	10.82 (5.39)
Prefer not to say	0 (0.00)	Anxiety subscale	14.72 (6.58)
*Country of birth*,* n (%)*		PTQ-C (6-item adapted version)^2^	13.63 (4.85)
Australia	55 (96.49)	CRQ-PA^2^	21.93 (5.97)
United Kingdom	1 (1.75)	PedsQL-SF^2,3^	78.42 (11.80)
Fiji	1 (1.75)	Physical functioning subscale^3^	84.91 (14.13)
*Current/prior mental health diagnosis*,* n (%)*		Emotional functioning subscale^3^	71.93 (15.72)
Generalised anxiety disorder	22 (38.60)	Social functioning subscale^3^	81.73 (17.63)
Attention deficit hyperactive disorder	17 (29.82)	School functioning subscale^3^	73.39 (20.38)
Depression	8 (14.04)		
Social anxiety disorder	8 (14.04)		
Separation anxiety disorder	4 (7.02)		
Obsessive compulsive disorder	3 (5.26)		
Eating disorder	1 (1.75)		
Conduct disorder	1 (1.75)		
Panic disorder	1 (1.75)		
Post-traumatic stress disorder	1 (1.75)		
Bipolar disorder	0 (0.00)		
Schizophrenia	0 (0.00)		
Unsure	4 (7.02)		
None of the above	24 (42.11)		

^1^Most parents in the sample completed the parent survey with respect to an adolescent who did not take part in this study (i.e., did not complete the adolescent-report survey)

^2^CRQ-PA: Co-Rumination Questionnaire—Parent-Adolescent; PedsQL-SF: Pediatric Quality of Life Inventory—short Form; PTQ-C: Bespoke Perseverative Thinking Questionnaire—Child Version (6 out of 15 items from child-report version); RCADS-25: Revised Children’s Anxiety and Depression Scale-25

^3^Reversed-scored

### Questionnaires

#### Mental health and wellbeing

Descriptive results for adolescent responses to mental health and wellbeing questionnaires are shown in Table 1. On the RCADS-25, the mean total score was 34.04 (SD = 12.74), with a depression subscale mean of 14.89 (SD = 6.07) and an anxiety subscale mean of 19.15 (SD = 7.97). These were markedly higher than symptom levels in community samples (e.g., [52]). On the SWEMWBS, the mean score was 18.40 (SD = 2.97), indicating moderate wellbeing (scale range = 7–35). On the PedsQL-SF, the mean score was 51.09 (SD = 16.92), indicating low-to-moderate quality of life (scale range = 0–100). For all of these non-RNT-specific measures, results were comparable across age and gender groups.

Descriptive results for parent-report responses to mental health and wellbeing questionnaires are shown in Table 2. As noted above, most parents in the sample completed the parent survey with respect to an adolescent who did not take part in this study (i.e., did not complete the adolescent-report survey). Specifically, only *n* = 16 parent-adolescent dyads participated (i.e., completed both parent-report and adolescent-report surveys). On the RCADS-25, the mean total parent-report score was 25.54 (SD = 10.53), with a depression subscale mean of 10.82 (SD = 5.39) and an anxiety subscale mean of 14.72 (SD = 6.58). These means are consistent with clinical samples and markedly higher than symptom levels in non-clinical samples (e.g., [44]). On the PedsQL-SF, the mean score was 78.42 (SD = 11.80), indicating moderate-to-high quality of life. On all non-RNT-specific measures, results were similar across gender groups. Given the small number of parent-report responses for older adolescents (12%), aged-based comparisons of means were not conducted.

#### RNT and co-rumination

Adolescents reported high levels of RNT and co-rumination (Table 1). On the PTQ-C, the mean score was 39.00 (SD = 10.67), comparable to clinical samples [53] and higher than community samples [45]. On the CRQ-SF, the mean score was 21.06 (SD = 7.51) and on the CRQ-PA, the mean score was 19.59 (SD = 8.10), both consistent with community samples [46, 54]. Mean scores on all three RNT-specific measures were similar across younger and older adolescents. The pattern of means suggest that scores on the PTQ-C were higher for non-binary adolescents (M = 46.29) than for their female (M = 38.94) or male (M = 35.80) peers, but no other marked differences in RNT-specific mean scores were observed across gender groups.

Parents reported that their adolescent children experienced moderate levels of RNT and co-rumination (Table 2). On the adapted 6-item parent-report version of the PTQ-C, the mean score was 13.63 (SD = 4.85). The mean score on the CRQ-PA was 21.93 (SD = 5.97), consistent with reports in community samples [46]. Mean scores on both measures were similar across females and males. Given the small number of parent-report responses for older adolescents (12%), aged-based comparisons of means were not conducted.

### Understanding and experiences of RNT

#### Definitions of worry and rumination

Adolescent definitions of worry and rumination are outlined in Table 3. Adolescent definitions of worry referenced four main themes: emotional discomfort (71%; e.g., unease, stress, anxiety), thinking with a negative focus or consequence (29%), repetitive, excessive, or stuck thinking (26%), and physical symptoms of anxiety (10%).

In contrast to these comprehensive definitions of worry, only 61% of older adolescents and 19% of younger adolescents had heard of rumination prior to completing the survey (Table 4). When asked to define rumination or guess what it meant (Table 3), over a quarter of all adolescents (28%) said they were unsure or provided non-RNT related definitions (e.g., spreading rumours). Nonetheless, adolescent definitions of rumination also referenced repetitive, excessive, or stuck thinking (47%) and thinking with a negative focus or consequence (35%). A small proportion of adolescents referenced deep or focused thinking (7%) and other thought-related definitions (4%, e.g., reminiscing, anticipatory thinking).

**Table 3:** Adolescent definitions of worry and rumination

Survey topics	Themes	*n* (%)	Example responses
Definition of worry	Emotional discomfort	51 (70.83)	“A general sense of unease with a slight sense of dread”
	Thinking with negative focus or consequence	21 (29.17)	“Think something bad is happening… stops you from doing or thinking about anything else”
	Repetitive, excessive, or stuck thinking	19 (26.39)	“Thinking about the same thing when there is nothing/little to be done to change it”
	Physical symptoms of anxiety	7 (9.72)	“Feeling like you struggle to breath, pressure, increased heart rate”
	Other thought-related definitions	3 (4.17)	“Wondering what will happen next”
Definition of rumination	Repetitive, excessive, or stuck thinking	34 (47.22)	“Constant, recurring thoughts that go in circles”
	Thinking with negative focus or consequence	25 (34.72)	“Worrying that prevents you from doing normal everyday activities”
	Unsure or non-RNT-related definitions	20 (27.78)	“Rumours being made?”
	Deep or focused thinking	5 (6.94)	“Deep thought about something”
	Other thought-related definitions	3 (4.17)	“Reminiscing of the past?”

#### RNT frequency and duration

As shown in Table 4, adolescent participants most commonly reported worrying and/or ruminating several times a day (26.9% and 35.6% of younger and older adolescents respectively), followed by daily (19.2% and 20.0% of younger and older adolescents respectively). Younger adolescents tended to report slightly lower RNT duration than older adolescents. Younger adolescents most commonly reported worrying/ruminating for 20–30 min (26.9%), followed by 10–20 min (23.1%) or 30 min–1 h (23.1%) on each occasion. Older adolescents most commonly reported worrying/ruminating for 30 min–1 h (31.1%) followed by 20–30 min (17.8%) on each occasion.

**Table 4:** Adolescent Understanding and experience of RNT

Survey questions & response options	Younger adolescent-report *N* = 26 *n* (%)	Older adolescent-report *N* = 46 *n* (%)	Parent-report (All Ages) ^4^ *N* = 57 *n* (%)
*Have you heard of ‘rumination’?*			
Yes	5 (19.23)	28 (60.87)	
No	21 (80.77)	18 (39.13)	
*What time of the day are you (is your child) most likely to worry or ruminate* ^*1*^ *? Please choose all that apply*			
Morning	10 (38.46)	12 (26.09)	18 (31.60)
Afternoon	9 (34.62)	20 (43.48)	15 (26.30)
Evening	11 (42.31)	27 (58.70)	33 (57.90)
Late at night / in bed	20 (76.92)	35 (76.09)	37 (64.90)
*What do you (does your child) usually worry / ruminate about? Please choose all that apply*			
Past events	25 (96.15)	43 (93.48)	42 (73.68)
Future events	18 (69.23)	36 (78.26)	41 (71.93)
Schoolwork / assignments / exams	14 (53.85)	34 (73.91)	25 (43.86)
Things I don’t (he/she doesn’t) like about myself (him/herself)	14 (53.85)	28 (60.87)	24 (42.11)
Friendships	13 (50.00)	34 (73.91)	25 (43.86)
Why things have happened to me (him/her)	13 (50.00)	28 (60.87)	22 (38.60)
Other family problems	12 (46.15)	18 (39.13)	13 (22.81)
Social media posts / comments / reactions	12 (46.15)	10 (21.74)	9 (15.79)
How I (he/she) feel(s)	11 (42.31)	27 (58.70)	33 (57.89)
How I (he/she) would cope if certain things were to happen	11 (42.31)	26 (56.52)	16 (28.07)
Family relationships	9 (34.62)	23 (50.00)	13 (22.81)
Why I (he/she) feel(s) a certain way	8 (30.77)	20 (43.48)	18 (31.58)
My (his/her) health	6 (23.08)	24 (52.17)	10 (17.54)
Romantic relationships	3 (11.54)	15 (32.61)	4 (7.02)
World events / the news	3 (11.54)	10 (21.74)	8 (14.04)
Other	3 (11.54)	1 (2.17)	7 (12.28)
Unsure	0 (0.00)	0 (0.00)	1 (1.75)
*When you get stuck in negative thoughts (like rumination/worry)*,* what does it usually feel like?*			
Mainly pictures/videos in my head, very few words	0 (0.00)	0 (0.00)	
More pictures/videos than words in my head^2^	5 (20.00)	2 (4.76)	
An equal mix of pictures/videos and words in my head	4 (16.00)	7 (16.67)	
More words than pictures/videos in my head^2^	6 (24.00)	17 (40.48)	
Mainly hearing /saying words in my head, very few pictures/videos	10 (40.00)	16 (38.10)	
*Do you tend to worry/ruminate more or less when you are with other people?*			
I worry/ruminate more when I am with others	6 (23.08)	4 (8.89)	
I worry/ruminate less when I am with others	20 (76.92)	41 (91.11)	
*On average*,* how often do you find yourself worrying/ruminating?*			
Constantly	3 (11.54)	7 (15.56)	
Several times a day	7 (26.92)	16 (35.56)	
Daily	5 (19.23)	9 (20.00)	
More than half the days a week	5 (19.23)	7 (15.56)	
Weekly	3 (11.54)	3 (6.67)	
Fortnightly	1 (3.85)	3 (6.67)	
Monthly	1 (3.85)	0 (0.00)	
Every couple of months or so	1 (3.85)	0 (0.00)	
*When you worry or ruminate*,* how long do you usually spend worrying/ruminating?*			
Less than 5 min	0 (0.00)	0 (0.00)	
5–10 min	3 (11.54)	5 (11.11)	
10–20 min	6 (23.08)	6 (13.33)	
20–30 min	7 (26.92)	8 (17.78)	
30 min–1 h	6 (23.08)	14 (31.11)	
1–2 h	2 (7.69)	7 (15.56)	
More than 2 h	2 (7.69)	5 (11.11)	
*When I have lots of thoughts at night*,* I usually feel that I cannot control all these thoughts that I am having.*			
Yes	22 (84.62)	40 (88.89)	
No	4 (15.38)	5 (11.11)	
*Do you regularly have trouble falling asleep at night because you can’t stop worrying or ruminating?*			
Yes	15 (57.69)	29 (64.44)	
No	11 (42.31)	16 (35.56)	
*If yes*^*3*^, *what do you think or worry about? Choose all that apply.*			
Bad things that happened during the day	13 (50.00)	23 (50.00)	
Things I don’t like about myself	10 (38.46)	17 (36.96)	
Schoolwork problems	8 (30.77)	20 (43.48)	
Friendship problems	7 (26.92)	20 (43.48)	
Bad things that might happen tomorrow	6 (23.08)	21 (45.65)	
Family problems	5 (19.23)	15 (32.61)	
Relationship problems	1 (3.85)	11 (23.91)	

^1^Participants were given the following information as part of this question: “In this questionnaire, we use the word “ruminate” to mean thinking about the same things over and over, even though this thinking makes you feel bad.”

^2^These response options were not actually shown to participants (they were left empty and only the ends and middle of sliding scale were labelled)

^3^“If yes” refers to answer to previous question: “Do you regularly have trouble falling asleep at night because you can’t stop worrying or ruminating?”

^4^Parent-report data was only collected for aspects of adolescent RNT which were deem plausibly observable by parents. For the corresponding survey items, the wording of the parent-report question has been incorporated in parentheses in Column 1 of this table. Parent-report data was not segmented by adolescent age group since only a small minority (12%) of these responses were in relation to older adolescents

#### RNT content and triggers

The most common topics of RNT were consistent between younger and older adolescents, with RNT being predominantly past-focused in both age groups. As shown in Table 4, younger adolescents reported worrying and/or ruminating about past events (96%), future events (69%), schoolwork (54%), things they dislike about themselves (54%), friendships (50%), and why things happened to them (50%). Similarly, older adolescents reported worrying and/or ruminating about past events (93%), future events (78%), friendships (74%), schoolwork (74%), things they dislike about themselves (61%), and why things happened to them (61%). Several noteworthy discrepancies in common RNT topics were also observed across adolescent age groups. Specifically, younger adolescents were more likely to worry and/or ruminate about social media interactions than older adolescents (46% vs. 22%). However, compared to their older peers, younger adolescents were less likely to worry and/or ruminate about their health (23% vs. 52%), friendships (50% vs. 74%), romantic relationships (12% vs. 33%), schoolwork (54% vs. 74%), and how they feel (42% vs. 59%).

The majority of younger (64%) and older (79%) adolescents reported that their RNT took more of a verbal than visual form. However, some of both the younger (36%) and older adolescents (21%) reported that their RNT was equally verbal and visual or took more of a visual than verbal form.

Nighttime and being alone emerged as common triggers for RNT. Both younger and older adolescents were increasingly likely to engage in RNT as the day progressed, with RNT likelihood highest at night (76–77%). Only a minority of younger (39%) and older (26%) adolescents reported likely engagement in RNT in the morning. Similarly, parents reported the likelihood of their adolescent child engaging in RNT increasing throughout the day, from morning (32%) to night (65%). Consistent with these results, adolescents in both age groups reported inability to control RNT at night (younger adolescents: 85%; older adolescents: 89%) and regularly having trouble falling asleep as a result (younger adolescents: 58%; older adolescents: 64%). Being alone with one’s thoughts may be a key reason for higher rates of RNT at night compared to earlier in the day, since the majority of younger (77%) and older (91%) adolescents reported being less likely to engage in RNT when they are with others than when alone.

Free-text responses provided further insights into triggers (both internal and external) of adolescent RNT (Table 5). Among external (environmental) triggers, the most common were being alone or in a quiet environment, including bedtime (19%), interpersonal or social triggers (18%), and reminders of negative events or worries (11%). Among internal triggers, the most common were somatic changes (21%), particularly physical symptoms of anxiety (13%). Importantly, almost half of adolescent participants (46%) reported that they were unsure what triggered their RNT or that the triggers were random.

Parents also reported both external and internal triggers for their adolescent child’s RNT (Table 5). The most common external triggers were interpersonal or social triggers (32%) and school-related triggers (21%). The most common internal trigger was fatigue (14%). Over a quarter (26%) of parents were unsure what triggered their adolescent child’s RNT or reported that the triggers were random.

**Table 5:** RNT triggers

Survey topics	Themes	*n* (%)	Example responses
RNT triggers (adolescent perspective)	External triggers		
	Being alone or in a quiet environment	14 (19.44)	“When everything is quiet and after I turn my music off”
	Interpersonal or social trigger	13 (18.06)	“Having a fight with a friend”
	Reminder of negative events or worries	8 (11.11)	“If someone says or does something that reminds me of a negative memory”
	Other external triggers	6 (8.33)	“If there is a change in plans”
	Negative events (general)	5 (6.94)	“Bad things happening around me (if it affects me) locally/worldwide”
	School-related triggers	3 (4.17)	“Upcoming tests I feel unprepared for”
	Internal triggers		
	Somatic changes	15 (20.83)	“Either my breathing starts to go funny leading to headaches”
	Emotional discomfort	3 (4.17)	“I get uneasy and unsettled”
	Mental exertion or confusion	2 (2.78)	“Mental exertion of any kind”
	Thinking about the past	2 (2.78)	“Dread (I’ve made a mistake)”
	Unsure or random	33 (45.83)	“Unsure, it just sort of starts”
RNT triggers (parent perspective)	External triggers		
	Interpersonal or social trigger	18 (25.00)	“Navigating friendships / Other kids being mean”
	Other external triggers	14 (19.44)	“The unknown”
	School-related triggers	12 (16.67)	“Pressure to go to school”
	Daily stressors	8 (11.11)	“Busy schedule”
	Changes to routines or plans	6 (8.33)	“Changes - particularly to plans and routines”
	Negative events	6 (8.33)	“After she’s had a negative experience”
	Being alone or in a quiet environment	5 (6.94)	“Lying in bed with nothing to distract his thoughts”
	Internal triggers		
	Other internal triggers	11 (15.28)	“Hormones (menstrual cycle)”
	Fatigue	8 (11.11)	“When tired – poor sleep leading in”
	Worrying	4 (5.56)	“Thinking about upcoming events and worrying about things that might happen”
	Unsure or random	15 (20.83)	“Unsure - happens a lot!”

#### RNT circuit breakers and reduction strategies

Adolescents and parents reported a range of ‘circuit-breakers’ that typically stop RNT (Table 6). The most common adolescent-report circuit-breakers were distractions (47%; e.g., hobbies, focusing on another task) and interacting with others (42%). Other common circuit-breakers were calming/soothing activities or stimuli (18%) and rest or reducing demands (15%). The most common parent-report RNT circuit breakers for their adolescent child were interacting with others (51%) and distractions (32%). However, 11% of adolescents and 29% of parents were unsure what stops adolescents’ RNT or reported that nothing stops it.

When asked about RNT-reduction strategies, 43% of adolescents reported that there is nothing they currently do to stop their RNT. Similarly, 35% of parents reported that there is nothing their adolescent child does to stop their RNT. However, a range of RNT-reduction strategies were also reported. The most common strategies adolescents used to stop RNT were engaging in distraction activities (36%). Additional strategies included interacting with others (11%) and breathing or grounding techniques (7%). The most common strategy that parents reported that their adolescent child uses to stop RNT was also distraction (39%), followed by interacting with others (16%) and engaging in relaxation or breathing techniques (9%). A full list of reported RNT reduction strategies can be found in Table 6.

**Table 6:** RNT circuit breakers and reduction strategies

Survey topics	Themes	*n* (%)	Example responses
RNT circuit-breakers (adolescent perspective)	Distractions	34 (47.22)	“Music, reading/watching comfort books/movies, patting my cat”
	Interacting with others	30 (41.67)	“Talking to friends and family”
	Calming or soothing activities	13 (18.06)	“Grounding myself”
	Rest or reducing demands	11 (15.28)	“No commitments for rest of day”
	Other	5 (6.94)	“Telling myself I am doing well and everything is okay”
	Unsure	5 (6.94)	“I don’t know, it usually takes a while”
	Moving to a new environment or withdrawal	4 (5.56)	“Getting away from people”
	Nothing	3 (4.17)	“Nothing: (“
	Problem resolution	3 (4.17)	“To complete the task or get a certain answer”
	Professional support	2 (2.78)	“Talking to my therapist”
RNT circuit-breakers (parent perspective)	Interacting with others	29 (40.28)	“Sometimes a hug and a good talk about ways to solve the problem”
	Distraction	18 (25.00)	“Distraction or refocus on some other task if possible”
	Unsure	14 (19.44)	“Unsure”
	Other	13 (18.06)	“Time”
	Calming or soothing activities	6 (8.33)	“Salt/calming bath”
	Increasing structure or predictability	6 (8.33)	“Increasing predictability or providing more structure/scaffolds”
	Rest or reducing demands	6 (8.33)	“Going to bed”
	Nothing	2 (2.78)	“Nothing”
	Professional support	2 (2.78)	“Medical support”
RNT reduction strategy (adolescent perspective)	None	31 (43.06)	“No”
	Distraction	26 (36.11)	“Sometimes playing games online helps, or playing with my dog, but not all the time”
	Interacting with others	8 (11.11)	“Text someone to distract myself”
	Breathing or grounding	5 (6.94)	“Grounding myself”
	Self-talk	4 (5.56)	“I tell myself that the thought is not real”
	Professional support	2 (2.78)	“Call a helpline”
	Unsure	2 (2.78)	“I don’t know”
	Sleep	1 (1.39)	“I’ll often just lie down and sleep”
RNT reduction strategy (parent perspective)	Distraction	22 (30.56)	“Going on a device”
	None	20 (27.78)	“N/a”
	Other	10 (13.89)	“She needs to withdraw until she processes it”
	Interacting with others	9 (12.50)	“He struggles to stop it himself. He relies on me to help him circuit break”
	Relaxation or breathing techniques	5 (6.94)	“Guided meditations or progressive muscle relaxation can help”
	Unsure	1 (1.39)	“Not sure”

#### Perceived benefit/purpose of RNT

When asked in what ways RNT helps them, most adolescents responded that RNT was unhelpful (56%) or were unsure how it helps them (17%). Similarly, when asked what purpose RNT may serve for their child, a sizeable proportion of parents stated that RNT had no purpose (30%) or were unsure what purpose it served (18%). Among the minority of adolescents who did perceive some benefit to their RNT, responses referenced preparation or self-protection (15%) and processing experiences or gaining insight (13%). Similarly, some parents reported that RNT helps their adolescent child process experiences or gain insights (26%) or gain a sense of control or self-protection (16%). A full list of adolescent- and parent-report responses can be found in Table 7.

**Table 7:** Perceived benefit/purpose of RNT

Survey topics	Themes	*n* (%)	Example responses
RNT benefit (adolescent perspective)	None	40 (55.56)	“It doesn’t help me”
	Unsure	12 (16.67)	“I don’t know”
	Preparation or self-protection	11 (15.28)	“It makes me more prepared for all scenarios”
	Process experiences or gain insights	9 (12.50)	“A little bit helps identify problems and solutions”
	Increase motivation or action	3 (4.17)	“With schoolwork it helps me get it done”
	Increase consideration for others	1 (1.39)	“It means I’m very thoughtful of how others feel”
RNT purpose (parent perspective)	None	17 (23.61)	“No purpose, he just struggles to switch off his brain”
	Process experiences or gain insights	15 (20.83)	“A way of processing worries about things they can’t control or they don’t know about”
	Unsure	10 (13.89)	“Not sure but it is hugely time consuming”
	Sense of control or self-protection	9 (12.50)	“We know it’s his body trying to keep him safe”
	Communicate distress or signal needs	6 (8.33)	“Communicating that he needs help/wants closeness”
	Problem-solving	4 (5.56)	“Tries to solve the problems”

#### Benefits of RNT reduction

Adolescents and parents reported a range of anticipated potential benefits of adolescent RNT reduction in terms of both improved daily functioning and emotional wellbeing (Table 8). The most common benefits noted by adolescents were decreasing tension, stress, or anxiety (31%), increasing happiness or enjoyment (28%), improving sleep, fatigue, or energy (19%), improving thinking, clarity, or focus (19%), and increasing engagement in valued actions (19%). Similarly, the most common parent reported benefits for their adolescent child were decreasing tension, stress, or anxiety (37%), increasing happiness, enjoyment, or positivity (32%), increasing engagement in valued actions (23%), increasing confidence or self-esteem (18%), and improving relationships or social functioning (18%). A full list of adolescent- and parent-report responses can be found in Table 8.

**Table 8:** Benefits of RNT reduction

Survey topics	Themes	*n* (%)	Example responses
RNT reduction benefit (adolescent perspective)	Improve emotional wellbeing		
	Decrease tension, stress, or anxiety	22 (30.56)	“I would be able to relax more I would not worry as much”
	Increase happiness or enjoyment	20 (27.78)	“I would enjoy the fun times much more”
	Increase calm, peace, or acceptance	9 (12.50)	“I wouldn’t have anxiety and would simply let things be the way they were”
	Improve mental health (general)	5 (6.94)	“Would have much better mental health”
	Increase confidence or self-esteem	5 (6.94)	“I would feel more confident to do things that I want to do”
	Be more present	4 (5.56)	“I would be more present in the moment”
	Improve daily functioning		
	Improve sleep, fatigue, or energy	14 (19.44)	“Allow me to sleep better and have more energy”
	Improve thinking, clarity, or focus	14 (19.44)	“Could think straight. Less foggy”
	Increase engagement in valued actions	14 (19.44)	“It would allow me to take bold moves and bigger chances without worrying”
	Improve relationships or social functioning	6 (8.33)	“Way way happier and more relaxed in relationships w friends and partners i think”
	Improve school-related functioning	5 (6.94)	“Do better in school”
	Non-specific benefit	4 (5.56)	“So much better!!!!!!!”
	Unsure or no benefit	3 (4.17)	“I don’t know”
RNT reduction benefit (parent perspective)	Improve emotional wellbeing		
	Decrease tension, stress, or anxiety	21 (29.17)	“She would be easier going, less stressed and anxious”
	Increase happiness, enjoyment, or positivity	18 (25.00)	“Happier child”
	Increase confidence or self-esteem	10 (13.89)	“Increase self-esteem/self-worth
	Improve mental health (general or other)	8 (11.11)	“Less likely future mental health problems”
	Improve daily functioning		
	Increase engagement in valued actions	13 (18.06)	“Allow her to step forward into new opportunities rather than not do things out of fear”
	Improve relationships or social functioning	10 (13.89)	“Developing and maintaining friendships”
	Improve sleep, fatigue, or energy	8 (11.11)	“Better rest, better energy”
	Improve thinking, clarity, or focus	7 (9.72)	“Clearer thinking/problem-solving”
	Improve school-related functioning	4 (5.56)	“Greater participation in school”
	Improve appetite	2 (2.78)	“Increased appetite/ability to eat a wider range of foods”

## Discussion

Adolescence is a developmental window of vulnerability for the onset of common mental health challenges [1, 2]. The current mixed methods study explored the lived experience of RNT during adolescence, given evidence that this transdiagnostic process predicts the onset and maintenance of a range of mental disorders [27–29]. By combining quantitative and qualitative data from both adolescents and parents, the study has provided novel insights that can inform the design of age-appropriate RNT-focused preventive interventions for depression and anxiety. Findings broadly support and extend prior qualitative research on RNT in older adolescents and adults [35–38], while also revealing developmental differences that highlight the unique vulnerabilities and needs of younger adolescents struggling with RNT.

Mean RNT severity scores among both younger and older adolescents in our study were comparable to clinical samples [53], and substantially higher than typical community samples [45]. This may reflect elevated depression and anxiety in our sample, but also underscores that high levels of RNT can emerge as early as age 10. Furthermore, patterns of RNT frequency and duration among our community-recruited younger and older adolescents were comparable to those observed in clinically depressed older adolescent [36] and community adult samples [35], with most adolescents in our study reporting RNT episodes several times per day lasting between 20 min to an hour. Older adolescents reported slightly longer and more frequent RNT episodes than younger adolescents, indicating a potentially worsening age-related trajectory. Together, these findings suggest that early adolescence is not only a relevant developmental period in which to target RNT via interventions, but may also be an optimal time to introduce RNT management skills before maladaptive thought processes consolidate into habits [55]. Early targeting of RNT is crucial as higher RNT predicts earlier onset, greater severity, and higher likelihood of relapse in depressive and anxiety disorders [10].

Parallelling high levels of RNT, adolescents in our study reported elevated levels of co-rumination with both peers and parents, consistent with prior findings from community samples [46, 54]. Furthermore, parents also reported elevated levels of co-rumination with their adolescent children, again consistent with prior reports from community samples [46]. These findings warrant attention, given that co-rumination predicts heightened depressive and anxiety symptoms over time [40–42]. The current study confirms prior research suggesting that co-rumination is common even in early adolescence [41], and although speculative, it is possible that parents may inadvertently model or reinforce co-ruminative tendencies when communicating with their children [46]. The co-occurrence of elevated RNT and co-rumination observed in the current study highlight potential bidirectional relationships between these two transdiagnostic processes, and suggest that co-rumination may be an important target to incorporate in RNT-reduction programs.

When asked to demonstrate conceptual understanding of RNT, adolescents in our study showed comprehensive collective understanding of the multifaceted nature of worry, but limited familiarity with the concept of rumination. In line with findings from an adult sample [35], adolescent definitions of worry from our study referenced cognitive, emotional, and physiological aspects of worry. However, a significant proportion of adolescents were unfamiliar with the term “rumination”, with more than double the percentage of younger compared to older adolescents reporting not having previously encountered the concept. This difference may be due to linguistic or developmental differences between younger and older adolescents, and highlights the importance of tailoring language in RNT programs for early adolescents to avoid disengagement. Furthermore, adolescent definitions of rumination mostly referenced cognitive features (e.g., stuck thinking), in contrast to previous qualitative studies with older adolescents and adults, in which emotional features (e.g., distress) were more frequently referenced [36–38, 56]. The relative absence of emotional features from adolescent definitions of rumination in our sample may be reflective of their unfamiliarity with the concept. Nonetheless, adolescent definitions of worry and rumination in our sample showed considerable overlap in referencing repetitive, excessive, and negative thinking, supporting findings on the similarities between these two thought processes [57–59] and further strengthening the validity of targeting both processes simultaneously via the umbrella RNT construct.

Adolescent perspectives of RNT content revealed notable similarities and differences between younger and older adolescents. In both age groups, RNT content tended to be slightly more focused on past than future events, consistent with the findings from studies with adult samples [35, 37], although potential overlap between temporal orientations should also be acknowledged. On a more granular level, the same topics were endorsed as the most common content of worry and/or rumination across both age groups: schoolwork, friendships, things adolescents dislike about themselves, and why things happened to them. Nonetheless, several notable discrepancies in content were noted across adolescent age groups. Younger adolescents were more than twice as likely to worry and/or ruminate about social media interactions than their older peers, potentially reflecting their relative unfamiliarity with this mode of interaction or heightened self-consciousness during vulnerable periods [60–62]. In contrast, compared to their older peers, younger adolescents were markedly less likely to worry and/or ruminate about their health, friendships, romantic relationships, schoolwork, and how they feel, reflecting age-related differences in key stressors and potentially lower self-reflexivity in early adolescence. Nonetheless, substantial proportions of younger adolescents endorsed abstract, over-generalised styles of thinking as common features of their RNT (e.g., almost a third reported commonly worrying and/or ruminating about “why I feel a certain way”). Abstract thought content is a key feature of RNT [63, 64], and our findings suggest this may be detectable even during early adolescence.

Most adolescents in our study reported experiencing RNT in a predominantly verbal format, consistent with common conceptualisations of RNT and worry [11]. These conceptualisations propose that RNT is a verbal strategy that serves to suppress distressing mental imagery of threats [65]. However, our results suggest that this verbal dominance may be less pronounced in younger adolescents, with over a third of younger adolescents in our sample experiencing RNT as more visual than verbal or a roughly equal mix of verbal and visual content, compared to approximately a fifth of older adolescents reporting the same. Previous studies have also noted the presence of imagery in both adolescent and adult RNT [36, 37]. Together, these findings underscore the need for early adolescent RNT interventions to include strategies to reduce both verbal and imagery-based RNT.

Interpersonal and social events were commonly identified as external triggers of RNT by both adolescents and parents in our study, consistent with the findings of prior studies in adolescents and adults [35, 36, 38]. Although we did not explore the extent to which interpersonal RNT triggers experienced by our sample coincided with co-rumination, moderate levels of co-rumination were reported by younger and older adolescents as well as parents, in the context of both friend and parent-child interactions. Additionally, a prior study involving older adolescents identified co-rumination as a common trigger for RNT [36]. Together, these findings further underscore a need for targeted support in helping adolescents develop skills to manage co-rumination as a pathway to reducing RNT. In addition to external RNT triggers, somatic symptoms and fatigue were identified by adolescents and parents as common internal triggers for RNT. However, many adolescents, especially younger adolescents, were unable to identify specific RNT triggers, indicating the importance of psychoeducation and structured self-monitoring to help adolescents detect and respond to emerging RNT more effectively.

A further area warranting attention is the timing and context in which RNT tends to arise. Across both age groups, adolescents most commonly reported engaging in RNT at night or when alone, with likelihood of RNT engagement being lowest in the morning and increasing as the day progresses. These patterns are consistent with findings in adult populations [35] and suggest that the end of the day may represent a particularly vulnerable period for the onset or escalation of RNT. Interventions for early adolescents may therefore benefit from incorporating evening- and/or bedtime-specific coping strategies to help mitigate RNT during these high-risk periods. Such strategies are particularly important given evidence that rumination mediates the association between adolescent insomnia and depression [66], and adolescent sleep disturbance predicts increases in internalising symptoms via both general and pre-sleep RNT [29].

Distraction and social interaction were reported to frequently interrupt RNT, functioning both as passive circuit breakers (e.g., an external event capturing attention) and as active coping strategies (e.g., deliberately shifting focus to another activity). These findings are consistent with and extend upon prior adolescent and adult studies, in which participants also cited distraction and social interactions as common ways to stop RNT [35, 37].

Concerningly, almost half of the adolescents in our study reported not actively engaging in any strategies to reduce their RNT and that nothing stops their RNT. These rates are higher than those reported by adults [35], but echo other adolescent qualitative studies where participants described a sense of helplessness and inertia in the face of RNT [56].

Over half of the adolescents in our study reported that RNT was unhelpful or had no purpose, with only a minority citing benefits such as preparation, self-protection, or emotional processing. These findings are consistent with the predominantly negative views depressed adolescents expressed regarding their rumination in a previous qualitative study [36], but diverge from the findings of adult studies in which higher percentages of participants endorsed positive metacognitive beliefs regarding their RNT [35]. Since positive and negative metacognitions can both contribute to RNT [67], interventions could aim to help younger adolescents gain insight into the function of their RNT, as a way to help them develop adaptive strategies to manage their RNT.

The current mixed methods study provides a comprehensive and nuanced understanding of adolescent experiences of RNT by integrating quantitative and qualitative insights from both adolescent and parental perspectives. Findings from the current study form a solid foundation for the design of an engaging and developmentally appropriate transdiagnostic program to reduce RNT, co-rumination, and prevent depression and anxiety in early adolescence. Adolescents and parents described a range of anticipated benefits from reducing RNT, including improved mood, better sleep, increased mental clarity, and enhanced ability to engage in meaningful activities. These benefits can be powerfully leveraged to enhance program engagement among both adolescents and parents.

Several limitations of the current study should also be acknowledged. First, most adolescent and parent participants did not form matched dyads, which precluded direct comparison between adolescent self-reports and parental perceptions due to insufficient power, and limited the interpretability of parent-child findings in the current study. Future studies may benefit from examining within-dyad concordance to better understand divergence in experienced versus observed adolescent RNT. Second, although the study recruited from the general community, the sample was skewed towards a culturally homogenous and predominantly female cohort of adolescents with clinically elevated symptoms of depression and anxiety and self-identified neurodivergence, potentially limiting generalisability to other populations. The predominantly clinical and neurodivergent nature of our self-selecting sample may reflect a participation bias among adolescents and families with existing concerns about mental wellbeing. Third, although the study aimed to recruit broadly across both younger and older adolescents, our adolescent-report sample consisted of significantly more older than younger adolescents. Therefore, caution must be exercised when extrapolating insights from our findings to inform the design of early-adolescent-specific interventions. Fourth, given the cross-sectional and retrospective self-report nature of data collected, causal directionality between variables cannot be established and recall bias may limit the generalisability of findings. Fifth, the extent to which participants distinguished between the constructs of rumination and worry was not explored, and both quantitative and qualitative results may contain conceptual overlap between these constructs.

## Conclusion

The current study has provided valuable insights into how young people aged 10–18 perceive and manage RNT. RNT and the related phenomenon of co-rumination are commonly experienced in adolescence, from as early as age 10. RNT causes distress to young people often lacking in effective coping strategies and warrants further attention alongside co-rumination as transdiagnostic intervention targets in preventive mental health programs.

## Supplementary Information

Below is the link to the electronic supplementary material.

Supplementary Material 1. Perseverative Thinking Questionnaire – Child Version (Parent-Report). Parent-report questionnaire used in the current study to assess adolescent RNT, adapted from the previously published Perseverative Thinking Questionnaire – Child Version (child-report) [45].

Supplementary Material 2. Co-Rumination Questionnaire – Parent-Adolescent (Child-Report). Child-report co-rumination questionnaire used in the current study to assess co-rumination between parents and adolescent children, adapted from the previously published Co-Rumination Questionnaire – Mother-Adolescent [46].

Supplementary Material 3. Bespoke Repetitive Negative Thinking (RNT) Questionnaires. Bespoke adolescent- and parent-report qualitative RNT questionnaires used in the current study to explore adolescent understanding and experiences of RNT, adapted from a previously published bespoke adult self-report qualitative RNT questionnaire [35].

## Data Availability

The datasets used and/or analysed during the current study are available from the corresponding author upon reasonable request and subject to university ethical approvals.

## Abbreviations

CRQ-SF: Co-Rumination Questionnaire—Short Form
CRQ-MA: Co-Rumination Questionnaire—Mother-Adolescent
CRQ-PA: Co-Rumination Questionnaire—Parent-Adolescent
PedsQL-SF: Pediatric Quality of Life Inventory Short Form
PISCF: Participant Information Sheet and Consent Form
PTQ-C: Perseverative Thinking Questionnaire—Child Version
RFCBT: Rumination-focused cognitive behavioural therapy
RCADS-25: Revised Children’s Anxiety and Depression Scale-25
RNT: Repetitive negative thinking
SWEMWBS: Short Warwick-Edinburgh Mental Well-Being Scale
