# Supplementary material for: Repetitive negative thinking in adolescence: a mixed methods study
Source: Child Adolesc Psychiatry Ment Health. 2025 Dec 10;20:5. doi: 10.1186/s13034-025-01005-0 (PMC12801832; doi:10.1186/s13034-025-01005-0)
Supplement: Supplementary file 1 — Supplementary Material 1. Perseverative Thinking Questionnaire – Child Version (Parent-Report). Parent-report questionnaire used in the current study to assess adolescent RNT, adapted from the previously published Perseverative Thinking Questionnaire – Child Version (child-report) [45]. [file 13034_2025_1005_MOESM1_ESM.docx]

**Additional File 1**

**Bespoke Repetitive Negative Thinking (RNT) Questionnaires**

**Parent-report questionnaire**

| **Question** | **Response options** |
| --- | --- |
| 1. What time of the day is your child most likely to worry or ruminate? (In this questionnaire, we use the word "ruminate" to mean thinking about the same things over and over, even though this thinking makes you feel bad.) Please choose all that apply. | - Morning - Afternoon - Evening - Late at night / in bed |
| 2. What does your child usually worry/ruminate about? Please choose all that apply | - Friendships - Family relationships - Romantic relationships - Other family problems - Social media posts / comments / reactions - Past events - Schoolwork / assignments / exams - Upcoming social events - Things he/she should have said/done - Past mistakes - Past conversations or interactions with others - Future conversations or interactions with others - World events / the news - His/her health - Past negative experiences - How he/she feels - Why things have happened to him/her - How he/she would cope if certain things were to happen - Things that might happen in the future - Why he/she feels a certain way - Things he/she doesn't like about him/herself - Other [text response] - Unsure |
| 3. What usually triggers your child to start worrying/ruminating (i.e. what happens just before he/she starts thinking like this?). Please list all the triggers in the space provided. If you're unsure, please write 'unsure'. | Open-ended text response |
| 4. In general, what makes your child's worrying/ruminating stop? If you're unsure, please write 'unsure'. | Open-ended text response |
| 5. Is there anything that your child does to stop him/herself from worrying/ruminating? If yes, please describe what he/she does. If no, please write 'N/A'. | Open-ended text response |
| 6. What purpose do you think worrying / ruminating serves for your child? | Open-ended text response |
| 7. If your child could stop ruminating / worrying so much, what are the biggest benefits this would have on his/her life? | Open-ended text response |

**Adolescent-report questionnaire**

| **Question** | **Response options** |
| --- | --- |
| 1. In your own words, please write your personal definition of 'worry'. Please note there are no right or wrong answers. | Open-ended text response |
| 2. Have you heard of 'rumination'? | - Yes - No |
| 3. Please describe what you think 'rumination' means. If you are not sure, you can take a guess. | Open-ended text response |
| 4. What time of the day are you most likely to worry or ruminate? Please choose all that apply.  (In this questionnaire, we use the word "ruminate" to mean thinking about the same things over and over, even though this thinking makes you feel bad.) | - Morning - Afternoon - Evening - Late at night / in bed |
| 5. What do you usually worry/ruminate about? Please choose all that apply. | - Friendships - Family relationships - Romantic relationships - Other family problems (e.g., money, health of family members) - Social media posts / comments / reactions - Past events - Schoolwork / assignments / exams - Upcoming social events - Things I should have said/done - Past mistakes - Past conversations or interactions with others - Future conversations or interactions with others - World events / the news - My health - Past negative experiences - How I feel - Why things have happened to me - How I would cope if certain things were to happen - Things that might happen in the future - Why I feel a certain way - Things I don't like about myself - Other (please specify) - Unsure |
| 6. When you get stuck in negative thoughts (like rumination/worry), what does it usually feel like? | - Mainly pictures/videos in my head, very few words - An equal mix of pictures/videos and words in my head - Mainly hearing/saying words in my head, very few pictures/videos |
| 7. Do you tend to worry/ruminate more or less when you are with other people? | Five-point sliding scale interactive sliding scale with the following anchors:  1. Mainly pictures/videos in my head, very few words  3. An equal mix of pictures/videos and words in my  head  5. Mainly hearing/saying words in my head, very few  pictures/videos  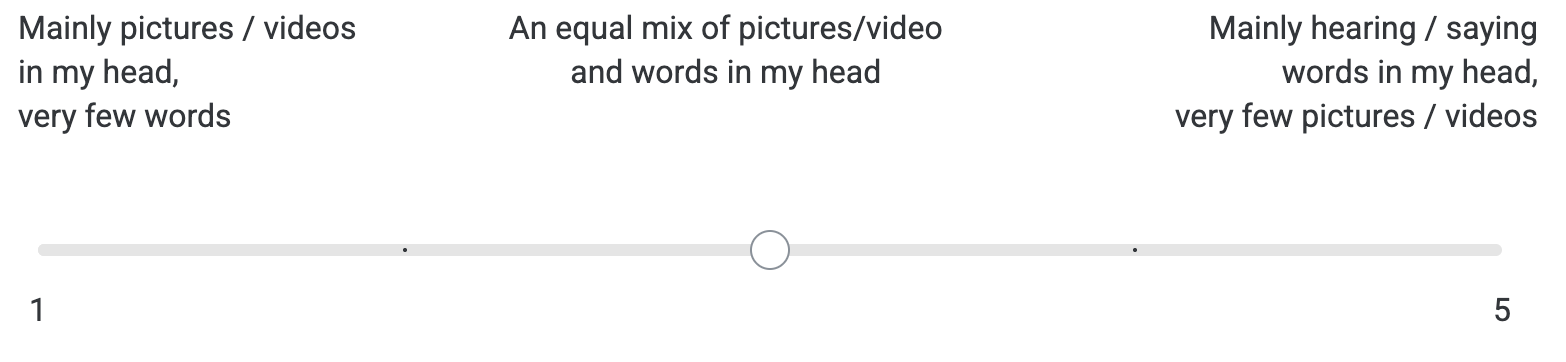 |
| 8. What usually happens just before you start worrying or ruminating about your problems? | Open-ended text response |
| 9. In general, what makes your worrying or ruminating stop? | Open-ended text response |
| 10. On average, how often do you find yourself worrying/ruminating? | - Constantly - Several times a day - Daily - More than half the days a week - Weekly - Fortnightly - Monthly - Every couple of months or more |
| 11. When you worry or ruminate, how long do you usually spend worrying/ruminating? | - Less than 5 minutes - 5-10 minutes - 10-20 minutes - 20-30 minutes - 30 minutes - 1 hour - 1-2 hours - More than 2 hours |
