# Supplementary material for: Repetitive negative thinking in adolescence: a mixed methods study
Source: Child Adolesc Psychiatry Ment Health. 2025 Dec 10;20:5. doi: 10.1186/s13034-025-01005-0 (PMC12801832; doi:10.1186/s13034-025-01005-0)
Supplement: Supplementary file 2 — Supplementary Material 2. Co-Rumination Questionnaire – Parent-Adolescent (Child-Report). Child-report co-rumination questionnaire used in the current study to assess co-rumination between parents and adolescent children, adapted from the previously published Co-Rumination Questionnaire – Mother-Adolescent [46]. [file 13034_2025_1005_MOESM2_ESM.docx]

**Additional File 2**

**Perseverative Thinking Questionnaire – Child Version (Parent-Report)**

In this questionnaire, you will be asked to describe how ***your child typically*** thinks about negative experiences or problems. Please read the following statements and rate the extent to which they apply to your child when he/she thinks about negative experiences or problems.

| **Question** | **Response options** |
| --- | --- |
| 1. My child thinks about many problems without solving any one of them. | 0 = Never  1 = Almost never  2 = Sometimes  3 = Often  4 = Almost always |
| 2. My child can't do anything else while thinking about his/her problems. |  |
| 3. When my child is thinking about certain things, he/she gets stuck and find it difficult to stop these thoughts. |  |
| 4. My child keeps asking him/herself questions without finding an answer. |  |
| 5. My child's thoughts prevent him/her from focusing his/her attention on other things. |  |
| 6. My child keeps thinking about the same things all the time. |  |
