# Supplementary material for: Repetitive negative thinking in adolescence: a mixed methods study
Source: Child Adolesc Psychiatry Ment Health. 2025 Dec 10;20:5. doi: 10.1186/s13034-025-01005-0 (PMC12801832; doi:10.1186/s13034-025-01005-0)
Supplement: Supplementary file 3 — Supplementary Material 3. Bespoke Repetitive Negative Thinking (RNT) Questionnaires. Bespoke adolescent- and parent-report qualitative RNT questionnaires used in the current study to explore adolescent understanding and experiences of RNT, adapted from a previously published bespoke adult self-report qualitative RNT questionnaire [35]. [file 13034_2025_1005_MOESM3_ESM.docx]

**Additional File 3**

**Co-Rumination Questionnaire - Parent-Adolescent (Child-Report)**

Think about the way you ***usually*** are with your Mum, Dad, or other parent/carer (whichever adult you usually talk to about your problems). Choose the option that best describes how you talk to each other.

| **Question** | **Response options** |
| --- | --- |
| 1. When I have a problem, my Mum/Dad and I talk to each other about it for a long time. | 1 = Not true at all  2 = A little true  3 = Somewhat true  4 = Mostly true  5 = Really true |
| 2. If I have a problem, my Mum/Dad  and I will spend our time together talking about it, no matter what else we could do instead. |  |
| 3. When I have a problem, my Mum/Dad always tries to get me to tell every detail about what happened. |  |
| 4. When my Mum/Dad and I talk about a problem that I have, we'll talk about every part of the problem over and over. |  |
| 5. When my Mum/Dad and I talk about a problem that I have, we talk a lot about the problem in order to understand why it happened. |  |
| 6. When my Mum/Dad and I talk about a problem that I have, we talk a lot about all of the different bad things that might happen because of problem. |  |
| 7. When my Mum/Dad and I talk about a problem that I have, we try to figure out everything about the problem, even if there are parts that we may never understand. |  |
| 8. When my Mum/Dad and I talk about a problem that I have, we spend a long time talking about how sad or mad I feel. |  |
